# Adapting the *in vitro* micronucleus assay (OECD Test Guideline No. 487) for testing of manufactured nanomaterials: recommendations for best practices

**DOI:** 10.1093/mutage/geae010

**Published:** 2024-03-19

**Authors:** Michael J Burgum, Clarissa Ulrich, Natascha Partosa, Stephen J Evans, Caroline Gomes, Svenja Berit Seiffert, Robert Landsiedel, Naveed Honarvar, Shareen H Doak

**Affiliations:** In Vitro Toxicology Group, Faculty of Medicine, Health and Life Sciences, Institute of Life Sciences, Swansea University Medical School, Singleton Park, Swansea, SA2 8PP, Wales, United Kingdom; BASF SE, Experimental Toxicology and Ecology, 67056, Ludwigshafen, Germany; BASF SE, Experimental Toxicology and Ecology, 67056, Ludwigshafen, Germany; In Vitro Toxicology Group, Faculty of Medicine, Health and Life Sciences, Institute of Life Sciences, Swansea University Medical School, Singleton Park, Swansea, SA2 8PP, Wales, United Kingdom; BASF SE, Experimental Toxicology and Ecology, 67056, Ludwigshafen, Germany; BASF SE, Analytical and Material Science, 67056, Ludwigshafen, Germany; BASF SE, Experimental Toxicology and Ecology, 67056, Ludwigshafen, Germany; Free University of Berlin, Pharmacy – Pharmacology and Toxicology, 14195 Berlin, Germany; BASF SE, Experimental Toxicology and Ecology, 67056, Ludwigshafen, Germany; In Vitro Toxicology Group, Faculty of Medicine, Health and Life Sciences, Institute of Life Sciences, Swansea University Medical School, Singleton Park, Swansea, SA2 8PP, Wales, United Kingdom

**Keywords:** Nanoparticles, micronucleus, WC/Co, OECD

## Abstract

The current Organisation for Economic Co-Operation and Development test guideline number 487 (OECD TG No. 487) provides instruction on how to conduct the *in vitro* micronucleus assay. This assay is one of the gold standard approaches for measuring the mutagenicity of test items; however, it is directed at testing low molecular weight molecules and may not be appropriate for particulate materials (e.g. engineered nanoparticles [ENPs]). This study aimed to adapt the *in vitro* micronucleus assay for ENP testing and underpins the development of an OECD guidance document. A harmonized, nano-specific protocol was generated and evaluated by two independent laboratories. Cell lines utilized were human lymphoblastoid (TK6) cells, human liver hepatocytes (HepG2) cells, Chinese hamster lung fibroblast (V79) cells, whole blood, and buffy coat cells from healthy human volunteers. These cells were exposed to reference ENPs from the Joint Research Council (JRC): SiO_2_ (RLS-0102), Au_5nm_ and Au_30nm_ (RLS-03, RLS-010), CeO_2_ (NM212), and BaSO_4_ (NM220). Tungsten carbide-cobalt (WC/Co) was used as a trial particulate positive control. The chemical controls were positive in all cell cultures, but WC/Co was only positive in TK6 and buffy coat cells. In TK6 cells, mutagenicity was observed for SiO_2_- and both Au types. In HepG2 cells, Au_5nm_ and SiO_2_ showed sub-two-fold increases in micronuclei. In V79 cells, whole blood, and buffy coat cells, no genotoxicity was detected with the test materials. The data confirmed that ENPs could be tested with the harmonized protocol, additionally, concordant data were observed across the two laboratories with V79 cells. WC/Co may be a suitable particulate positive control in the *in vitro* micronucleus assay when using TK6 and buffy coat cells. Detailed recommendations are therefore provided to adapt OECD TG No. 487 for testing ENP.

## Introduction

Engineered nanoparticles (ENPs) present a unique challenge to human hazard assessment and subsequently their regulation [1–4]. This is due to their small size, large surface area: volume ratio, and agglomeration dynamics. Paradoxically, it is these features (among others) of ENPs that give them their revolutionary properties in medical, industrial, and healthcare sectors [5,6]. The current regulatory situation for mutagenicity testing of ENPs is to follow the current OECD test guidelines (TGs). Testing the mutagenicity of ENPs includes a range of assays, which measure chromosomal breakage and gene mutagenicity, both *in vitro* and *in vivo*. The gold standard for measuring chromosomal damage *in vitro* is the micronucleus assay, which detects both clastogenic and aneugenic change and is described in detail by OECD TG No. 487. However, this assay is not optimal for evaluating the mutagenicity of ENPs due to their radically different physical properties as compared to small molecules, which the test is tailored towards. Small molecules can be readily dissolved in buffers or solvents, a trait not shared by the bulk of ENPs [7]. Other experimental aspects, such as the treatment (exposure) interval, suspension buffer, and dispersion protocol are examples of steps that need specific considerations when testing ENPs. For instance, the exposure time of ENPs has been greatly contested in the literature with the consensus being that pulse exposures of 1–3 hours do not offer enough time for the cells to internalize ENPs. It is much more likely that nano-induced cellular DNA damage may arise following > 24-hour exposures [1,5,8]. Since there is currently no official document to support the adaptation of the OECD TG No. 487 protocol for ENP testing, there is urgency in providing guidance on the necessary methodological changes required to facilitate a more robust and reliable assessment of the genotoxic potential of ENPs. This is necessary to better support the regulation of these materials and additionally, to reduce the repetition of inadequately performed mutagenicity studies [1]. Thus, in 2014, the OECD Genetic Toxicology Expert Group agreed that it was necessary to develop a nano-specific adaptation of OECD TG No. 487 (Genotoxicity of manufactured nanomaterials: Report of the OECD Expert Meeting, Series on the safety of nanomaterials no. 43). Initial work was performed by the European Commission’s Joint Research Centre (JRC) and published as technical reports, whereby reference ENPs were synthesized and extensively characterized for this purpose, coupled to dose-range finding cytotoxicity and uptake studies in five cell lines: A549, V79, TK6, CHO, and Caco-2 [9,10]. These studies provided the background data required to underpin the harmonization of a nano-adapted *in vitro* micronucleus assay protocol.

Prior to any toxicology testing, it is essential to achieve a stable suspension of ENPs, which requires dispersion in appropriate buffers. This is due to the tendency of ENPs to agglomerate in serum-containing cell culture media, resulting in exposure to larger agglomerates and inaccurate concentration information due to sedimentation [11]. The choice of suspension buffer, method of sonication, and subsequent dilution to working concentrations is laboratory-dependent as there are no set guidelines to follow, making it difficult to generate a toxicological consensus opinion on each material. In 2014, the probe sonication operating protocol was outlined in detail by the Horizon 2020 European Commission project NANoREG. This protocol explicitly states how ENPs in their raw state can be suspended in a bovine serum albumin (BSA) buffer and sonicated to produce a stable dispersion for 24 hours [12]. This approach is a possible way to unify laboratories under one common technique. To date, there have been a multitude of cell lines tested with the micronucleus assay which are not appropriate. Therefore, the suitability of the cell line must be carefully considered prior to regulatory testing; this has been highlighted in detail by Doak *et al*. and Elespuru *et al*. [1,5]. As detailed in OECD TG No. 487, TK6 human lymphoblastoid cells and buffy coat lymphocytes have been validated for use with the *in vitro* micronucleus assay when testing chemicals; extensive historical control data is available, they have a stable karyotype and demonstrate functional DNA repair capacity and P53 competency [13]. In addition to these two cell types, the OECD TG No. 487 describes several other cell lines that may be used for the *in vitro* micronucleus assay; however, whether all these cells can be applied for evaluating ENPs remains unknown.

The aim of this study was, therefore, to provide data to resolve several open questions required to adapt OECD TG No. 487 to reliably evaluate ENP mutagenicity *in vitro*. A harmonized protocol for the *in vitro* cytokinesis-blocked micronucleus (CBMN) assay was established as part of this approach, which was evaluated by two independent laboratories using two suspension cell lines and two adherent cell lines. The cells were exposed to ENPs of gold (Au; 5 nm and 30 nm), silicon dioxide (SiO_2_), cerium dioxide (CeO_2_), barium sulfate (BaSO_4_), and Tungsten carbide-cobalt (WC/Co) to determine ENP cytotoxicity and mutagenicity. Additionally, ENP-cellular interaction was assessed by laser ablation inductively coupled plasma mass spectrometry (LA ICP-MS), providing excellent sensitivity for nearly all elements of the periodic system and enabling direct analysis of cells and tissues on microscopic slides without time-consuming sample preparation.

## Materials and methods

### Preparation of the ENP test items

The RLS-03 & RLS-10 gold ENPs (Au_5nm_) & (Au_30nm_), respectively, RLS-0102 silicon dioxide (SiO_2_) ENPs were supplied by JRC and have been extensively characterized by Drewes *et al*. [9]. The JRC stock ENPs were sonicated in a 90W Ultrasonic Bath (Fisher Scientific #FB15046) for 20 minutes at 37°C to encourage destabilization of agglomerate material. Following sonication, working concentrations of particles were prepared in cell culture media using a 1:1 dilution for the highest concentration and using serial dilutions to prepare the rest of the dose range (Supplementary Table 1). Given each JRC ENP stock was at different concentrations, this process was material specific. Tungsten-carbide/cobalt (8 wt% WC/Co < 200 nm, 99.5% LOT#5561-072018, Nanostructured & Amorphous Materials Inc., USA), was used as a trial positive particulate control and was weighed, suspended, and sonicated according to the NANoREG protocol [12]. All dry powder ENPs were weighed using the OHAUS Explorer Semi-Micro Balance housed in a WAYSAFE (#GP1540). Cells were exposed to NM212 CeO_2_ and BaSO_4_ (NM220/(also identified as JRC NM50001a)) (Solvay, Lot#V106), which was handled and dispersed using the NANoREG protocol. A summary of the ENPs tested by which laboratory and in which cell lines can be found in Supplementary Table 1.

### Nanoparticle hydrodynamic diameter and surface potential characterization

The hydrodynamic diameter of the Au_5nm_, Au_30nm_, SiO_2,_ and WC/Co nanoparticles (at 20 µg/ml, 20 µg/ml, 100 µg/ml, and 100 µg/ml, respectively) were characterized in complete TK6 cell culture media by dynamic light scattering (DLS) and zeta potential using the Malvern Zetasizer Pro-Blue (Malvern Panalytical, UK) and software package ZS Explorer using Malvern ZEN 0040 low volume cuvettes. A total of three replicates were recorded each constituted of five runs each. The polydispersity index (PI) was also reported to provide an indicator of the width of the distribution within the data. The zeta potential was also characterized in complete TK6 cell culture media using the disposable Malvern Folded Capillary cell (#DTS1070) set to run at 37°C with three replicates per condition. The BaSO_4_ and CeO_2_ materials utilized in this study were characterized in a previous investigation, where their physico-chemical features were evaluated in both water and Dulbecco’s Modified Eagle Medium (DMEM) media containing FCS and were published in 2021 (Table 1) [14]. Substantial characterization of the JRC reference materials featured in Table 1 can be found in the technical report published online in 2018 by the JRC [9]. This report provides data sets pertaining to the stability of each material over a period of 0, 1, 4, 24, and 48 hours in Dulbecco’s Modified Eagle Medium: Nutrient Mixture F12 (F12) media + 10% calf-serum (CS), (DMEM) + 20% FBS, DMEM + 10% FBS and RPMI1640 + 10% horse serum (HS).

**Table 1.** Summary of the physico-chemical characteristics of the reference nanoparticles used in the study.

	Au_5nm_^a^ RLS-03	Au_30nm_^a^ RLS-10	SiO_2_^a^ RLS-01-02	WC/Co #5561HW	BaSO_4_^b^ NM-220	CeO_2_^b^ NM-212
Core composition	Gold	Gold	Silicon dioxide	Tungsten carbide-cobalt (8%.wt Co)	Barium Sulfate (Barite)	Cerium Dioxide (Cerianite)
Primary particle size by TEM (nm)	4.8	32	20	<200 nm	31.5	13.7
Morphology	Spherical	Spherical	Spherical	Irregular, some spherical	Irregular, some spherical	Irregular, edged, not spherical
Hydrodynamic diameter (nm). Data are presented as the mean ± the standard deviation	591.2 ± 99.4	720.2 ± 292.6	68.1 ± 6.5	1253 ± 434	41 ± 3 (at 10 µg/ml)	392 ± 116 (at 10 µg/ml)
PDI	0.4	0.5	0.5	0.76	1	0.5
Zeta potential (mV)	−11.6 ± 3.9	−4.81 ± 0.6	−11.4 ± 0.5	−10.3 ± 1.2	−11.6 ± 0.8	−10.7 ± 1.0
Surface area (m^2^/g)	n.t.	n.t.	n.t.	5–8	33.0	27.0

^a^Full physico-chemical characterization data has been published by the JRC in 2018 (9).

^b^Full physico-chemical characterization data was published in 2021 (14).

n.t., not tested.

The primary particle size and surface area of WC/Co nanoparticles was provided by the manufacturer (NanoAmor).

### Cell culture

#### Human lymphoblastoid (TK6), HepG2 and V79 cells

The TK6 cells (Catalogue number: 95111735) were purchased from the European Collection of Authenticated Cell Cultures (ECACC, UK). The cells were cultured in RPMI 1640 (ThermoFisher, UK #21870-076) supplemented with 10% Horse serum and 1% L-glutamine (Gibco, UK). TK6 cells were routinely sub-cultured for 2 weeks prior to testing; cells were regularly checked for potential changes to morphology and density by light microscopy. The human hepatocyte (HepG2) cell line was purchased from the American Type Culture Collection (ATCC, catalogue number; HB-8065). The cells were cultured in DMEM containing 10% FBS, 1% Penicillin/Streptomycin (P/S), and 1% L-glutamine (Gibco, UK). HepG2 cells were grown to 80% confluency before being routinely sub-cultured. The V79 cells (AbbVie GmbH) were cultured in MEM Eagle media (Pan-Biotech, UK) containing 1% l-glutamine, 1% Amphotericin, 10% FBS and 1% P/S. V79 cells could grow to 80% confluency before being routinely sub-cultured.

#### Whole blood cultures

For each experiment, fresh blood was collected from a single healthy donor not under medication and younger than 35 years. Prior to testing, the whole blood was diluted 1:10 in DMEM/Ham’s F12 (1:1) (DMEM/F12) medium containing stable glutamine supplemented with 10% [v/v] foetal calf serum (FCS), 1% Penicillin/Streptomycin (P/S), and 1% HEPES buffer (1M). For stimulation of the cells, 0.5% [v/v] Phytohemagglutinin (PHA, stock solution 0.6 mg/ml, final concentration 3 µg/ml) and 0.5% [v/v] sodium heparin (25 000 IE) was added. The cell suspension was cultured for 48 hours.

#### Buffy coat cultures

Buffy coat cells (containing white blood cells and platelets) were isolated from whole blood using the density centrifugation method with Ficoll-PaqueTM PLUS (GE Healthcare Bio-Sciences AB). The buffy coat cells were resuspended in RPMI 1640 medium containing stable glutamine supplemented with 20% [v/v] FCS and 1% Penicillin/Streptomycin (P/S) in a volume corresponding to a 1:5 dilution of the original blood volume. To stimulate the lymphocytes (contained within the buffy coat cells) specifically, PHA was added as described above for the whole blood cultures and cultured for 48 hours.

### Semi-automated in vitro cytokinesis-blocked micronucleus assay

#### TK6 & HepG2 cell cultures

TK6 (suspension cells) and HepG2 cells were seeded at 1.0 × 10^5^ cells/ml in T25 flasks along with satellite flasks per concentration to be counted for cytotoxicity and were then treated with each test ENP for 1 cell cycle (TK6 cell cycle time was approximately 13–15 hours, HepG2 cell cycle time was approximately 24 hours). Mitomycin-C (MMC, Merck #M4287) at 0.01 μg/ml was used as the molecular positive control, and WC/Co at 20 µg/ml and 100 µg/ml was tested as a positive particulate control. The *in vitro* CBMN assay was performed as described previously by Evans *et al*. and Burgum *et al*. [15,16]. On the day of exposures, cells are counted at least 2 hours before exposures (with suspension cells this can be done immediately prior to exposures). Cells were dosed with the ENPs prepared in cell-culture media, and the negative control being media only. Exposures were performed as close to sonication times as possible to avoid sedimentation. Following the exposure period, the satellite flasks were counted for the calculation of relative population doubling (RPD), a measure of cytotoxicity (Equation 1). Suspension cell counts were performed using a Beckman coulter counter by adding 100 µl of cells to 10 ml of diluent; adherent cells were counted with a Haemocytometer and trypan blue exclusion. Following the ENP exposure for 1 cell cycle, TK6 cells were centrifuged and washed with PBS in triplicate (removal of as much ENP as possible). V79 and HepG2 cells had exposure media aspirated and were then washed twice with PBS before being counted. TK6 cells were then re-seeded into clean T25 flasks in cell-culture media containing 3 µg/ml of cytochalasin B (cyto B, Merck #250233), and the cells were returned to the incubator for a further 1.5 cell cycles. V79 and HepG2 cells remained in the same flasks and were supplemented with fresh media containing the same concentration of cyto B.

#### V79 cell cultures

V79 (adherent) cells were seeded at 5.0 × 10^5^ cells/ml in T25 flasks (attachment period 24–28 hours) and were treated with each ENP along with satellite flasks per concentration to be counted for cytotoxicity and LA-ICP-MS assessment for 1 cell cycle (~14 hours). Ethyl methanesulfonate (EMS) at 500 μg/ml and 600 µg/ml was used as the chemical positive control, and WC/Co at 30 µg/ml and 100 µg/ml was tested as a potential positive particle control. After 1 cell-cycle (approximately 13–15 hours) test substance treatment, the cultures were rinsed twice with Hanks Balanced Salt Solution (HBSS). The cultures intended for mutagenicity assessment were incubated in MEM (incl. 10% [v/v] FCS) supplemented with cyto B (final concentration: 3 µg/ml; stock: 0.6 mg/ml in DMSO; AppliChem, Cat. No. A7657) for 24 hours. Cultures used for LA-ICP-MS assessments were trypsinized, fixed twice in methanol: acetic acid (19:1; −20°C), and spread on slides.

#### Whole blood cultures

After 48 hours, the activated cell cultures were pooled and centrifugated in 10 ml aliquots at 900*g* for 5 minutes. After centrifugation, the supernatant (culture medium) was removed, and the cells were suspended in ENP dilutions in the culture medium along with satellite tubes for each concentration for LA-ICP-MS assessment. All tubes were transferred into cell culture flasks and incubated for 20 hours. Mitomycin C (MMC; Roche Diagnostics) at 0.04 µg/ml and Colchicine (Col; Roche Diagnostics) at 0.05 µg/ml were used as the chemical positive controls, and WC/Co at 10, 30, 60, and 100 µg/ml were tested as potential positive particle control. At the end of the exposure period, the cells were transferred in tubes, centrifuged for 5 minutes at 900*g*, and resuspended in HBSS. Washing of the cells was repeated at least once. Then, the cells were centrifuged at (900*g*, 5 minutes) and resuspended in DMEM/F12 medium with 10% [v/v] FCS and transferred into 25 cm² cell culture flasks. Cyto B (final concentration: 6 µg/ml; stock: 2 mg/ml in DMSO; Merck, Cat. No. C2743) was added to the cultures intended for mutagenicity assessment and incubated at 37°C, 5% (v/v) CO_2_ and ≥ 90% relative humidity for 20 hours. To prepare the cells for the LA-ICP-MS assessments, they were separated from the ENPs using density centrifugation (Ficoll paque) and washed once at 900*g* for 5 minutes. The obtained cells were fixed twice with methanol: acetic acid (19:1; −20°C) and spread on slides.

#### Buffy coat cell cultures

After 48 hours, the activated cell cultures were pooled and centrifugated in 10 ml aliquots at 900*g* for 5 minutes. After centrifugation, the supernatant (culture medium) was removed, and the cells suspended in ENP dilutions in the culture medium along with a satellite tube for each concentration for LA-ICP-MS assessment. All tubes were transferred into cell culture flasks and incubated under agitation for 20 hours (corresponding to 1 cell cycle). Mitomycin C (MMC; Roche Diagnostics) at 0.04 µg/ml and Colchicine (Col; Roche Diagnostics) at 0.05 µg/ml were used as the chemical positive controls, and WC/Co at 10, 25 30, 60, and 100 µg/ml were tested as potential positive particle control. At the end of the exposure period, the cells were transferred in tubes, centrifuged for 5 minutes at 900*g*, and resuspended in HBSS. Washing of the cells was repeated at least once. Then, the cells were centrifuged at (900*g*, 5 minutes) and resuspended in RPMI medium with 20% [v/v] FCS and transferred into 25 cm² cell culture flasks. Cyto B (final concentration: 6 µg/ml; stock: 2 mg/ml in DMSO; Merck, Cat. No. C2743) was added to the cultures intended for mutagenicity assessment and incubated at 37°C, 5% (v/v) CO_2_ and ≥ 90% relative humidity for 20 hours. To prepare the cells for the LA-ICP-MS assessments, they were separated from the ENPs using density centrifugation (Ficoll paque) and washed once at 900*g* for 5 minutes. The obtained cells were fixed twice with methanol: acetic acid (19:1; −20°C) and spread on slides.

### Cell harvesting

#### Semi-automated approach

The TK6, HepG2, and V79 cells were harvested by centrifugation (230*g* for 5 minutes), resuspended in 5 ml of pre-warmed PBS, and centrifuged at 230*g* for 10 minutes. The supernatant was discarded; this was repeated a second time. The cells were then resuspended in hypotonic solution (KCl 0.56%), before being centrifuged immediately at 230*g* for 10 minutes. The cells were resuspended in Fixative 1 (methanol: acetic acid: NaCl (0.09%) (5:1:6 parts)) and incubated at 4°C for 10 minutes before centrifugation at 230*g* for 10 minutes. Cells were resuspended in Fixative 2 (methanol: acetic acid [5:1 parts]) and incubated at 4°C for 10 minutes before centrifugation at 230*g* for 10 minutes; this was repeated three times. Cells can be maintained overnight in Fixative 2 at 4°C, tubes covered by foil. The day before making slides, freshly opened microscope slides were placed in a glass tank of Fixative 2 at 4°C, for 2 hours before slide preparation (overnight). On the day of preparing slides, the fixative was replaced with dd.H_2_O. On the day of slide preparation, the fixed cell suspensions were centrifuged at 230*g* for 10 minutes and thoroughly re-suspended in ~1 ml of Fixative 2. Slides were removed from the dd.H_2_O and wiped dry with slide tissue. A total of 100 µl of the cell suspension was evenly pipetted onto the slide held at an angle. The slides were then stood vertically on tissue paper to dry. The cell density was checked to ensure cells were evenly distributed, without clumping. Once dried, the slides were stained with 30 µl of Vectashield mounting medium with DAPI, coverslip applied, and incubated in the dark for 15 minutes. Slides were scored using the Zeiss AxioCam HRc (Carl Zeiss Microscopy and Imaging, UK) semi-automated Metafer system. The details for the classifier used to support the analysis can be found in Supplementary Table 2. All experiments were performed in triplicate (*n* = 3) and 2000 binucleated (BN) cells per replicate were scored per concentration (6000 BN cells in total).


$$\begin{array}{l} RPD\;=\frac{No.\;of\;population\;doublings\;in\;treated\;cultures}{No.\;of\;population\;doublings\;in\;control\;cultures}\times \;100\; \end{array}$$ (1)

Where population doubling = [log (post-treatment cell number/initial cell number)]/log 2

All data have been presented as the Cell Viability as percentage (%). For RPD-calculated data; the RPD values were multiplied by 100 for a percentage for CBPI data sets, the % cytotoxicity was calculated from Equation 2 (above), then this value was subtracted from 100 to provide percentage cell viability.

#### Cell harvesting for manual scoring

After the cyto B treatment, the adherent cells were rinsed with pre-warmed HBSS and trypsinized. The obtained cell suspensions from all different cultures were treated in the same manner, namely the cells were centrifuged, and the cell pellet was treated with a hypotonic solution (10 minutes with 0.4% potassium chloride (KCl, Merck #1049360250) for V79 and buffy coat cells; 20 minutes with 0.28% KCl for whole blood cultures). After the hypotonic treatment, the cells were fixed twice by adding fixative (19 parts methanol and 1-part acetic acid: −20°C). Slides were prepared by immersing in deionized water followed by pipetting the fixed cells on the slide. The cells were stained with May-Grünwald (3 minutes) and 10% [v/v] Giemsa (in Titrisol, pH 7.2, 20 minutes) and mounted. The mutagenicity assessment was performed according to the OECD TG No. 487 guideline, namely by scoring a total of 2000 binucleated cells. The estimation of cytotoxicity for the human blood cells (whole blood cell cultures and buffy coat cells) was carried out according to the cytokinesis block proliferation index (CBPI, Equation 2) method described in the OECD TG No. 487 and depicted as the (%) cytotoxicity. For CBPI data sets, the (%) cytotoxicity was calculated from Equation 2, then this value was subtracted from 100 to provide percentage cell viability.


$$\begin{array}{l} \%\;Cytotoxicity=100-100\;\left(\frac{CBPI_{T}-1}{CBPI_{C}-1}\right), \end{array}$$ (2)

where T is treatment, C is control


$$\begin{array}{l} CBPI=\frac{No\;mononucleated\;cells\;+\;2\times BN\;cells\;+\;3\times MNCs}{N}, \end{array}$$

where N is the total number of cells scored

#### Cellular interaction assessment

For LA-ICP-MS analysis, a 193 nm ArF Excimer Laser (NWR193 Excimer Laser Ablation System, Elemental Scientific Lasers, Bozeman, MT USA) equipped with a two-volume cell (TwoVol^2^ Ablation Cell, Elemental Scientific Lasers) was coupled to an ICP-MS Triple Quadrupole (8900 ICP-MS Triple Quad, Agilent Technologies, Santa Clara, CA, USA). The ablated sample material was transported with a carrier gas flow (He, 800 ml/min) and introduced *via* a Dual Concentric Injector (DCI, Elemental Scientific Lasers) to the ICP-MS. An additional gas flow (Ar, 1 L/min) was added, and the sample material was transferred *via* a quartz injector pipe (inner diameter: 2.5 mm) into the plasma. The ICP-MS was equipped with a platinum sampler and skimmer. To resolve the issue of polyatomic interferences especially for low masses (e.g. ^31^P), it was operated in TQ modus with O_2_ as reaction gas. The set-up was tuned daily for maximum signal intensity and an oxide ratio (*m/z* 232/248) below 0.5% with a NIST Glass standard (NIST SRM 612, National Institute of Standards and Technology, Gaithersburg, MD, USA). Each cell was ablated separately with 50 bursts and a spot size of 25 µm. A laser pulse frequency of 100 Hz and a laser energy of 0.5 J/cm^2^ ensured a full ablation of the cells while avoiding ablation into the glass slide. The isotope ^31^P^16^O^+^ was monitored with a dwell time of 50 ms regardless of the type of experiment and chosen as internal standard. For Au nanoparticles (NPs) the isotope ^197^Au^+^, for CeO_2_ NPs the isotope ^140^Ce^16^O^+^ and for WC/Co NPs the isotopes ^59^Co^+^ and ^184^W^16^O^+^ were detected each with a dwell time of 50 ms. To distinguish a cell event from the continuous background, a threshold three times higher than the mean signal of the background was applied. For an identified cell, an association rate was calculated by dividing the summed signal intensities of the NP by the summed phosphorus signal intensities. This results in an association rate to what extent the NPs are allocated to the cells. This analysis could not be performed for SiO_2_ ENPs due to interference that occurs between the silica particles and the substrate material, which are glass microscope slides.

### Good practice

Methods were developed adhering to GIVIMP (guidance document on Good *In Vitro* Method Practices (OECD (2018), Guidance Document on Good *In Vitro* Method Practices (GIVIMP), OECD Series on Testing and Assessment, No. 286, OECD Publishing, Paris. https://doi.org/10.1787/9789264304796-en) by applying good scientific, technical, and quality practices in *in vitro* method development and method implementation.

### Statistics

All TK6 and HepG2 data are presented as the mean ± the standard deviation (SD). Statistical analysis was performed in GraphPad Prism software version 8.4.3 (Graphpad, USA). A one-way analysis of variance (ANOVA) was performed with post hoc Dunnett’s multiple comparisons applied to evaluate pairwise statistical significance between control and concentrations; the alpha value was set to 0.05. V79, whole blood and buffy coat cell cultures were analysed according to the proportion of cells containing micronuclei for each test group. A comparison of the micronucleus rates of each test group with the concurrent vehicle control group was carried out for the hypothesis of equal proportions (i.e. one-sided Fisher’s exact test, BASF SE). In addition, a statistical trend test (SAS procedure REG) was performed to assess a possible dose-related increase of micronucleated cells. The dependent variable was the number of micronucleated cells, and the independent variable was the concentration. It was tested to see if the slope was significantly different from zero. The trend was judged as statistically significant when *P* ≤ .05.

## Results

### Physico-chemical characterization

A summary of the physico-chemical features of the nanoparticles used in this study has been presented in Table 1. The primary nanoparticle size and morphology of the Au_5nm_, Au_30nm_, and SiO_2_ has been sourced from the JRC published report, while the BaSO_4_ and CeO_2_ characterization data was obtained from a 2021 study by Llewellyn *et al*. [9,14]. To ascertain the hydrodynamic diameter and zeta potential of the JRC reference nanoparticles, they were diluted down to exposure concentrations (detailed in the Methods) in complete TK6 cell culture media. Of the two types of AuNPs, the Au_5nm_ formed slightly smaller agglomerates of 591.2 nm when compared with the Au_30nm_ agglomerate size of 720.2 nm. Given the polydispersity index (PDI) for both AuNPs was 0.4 and 0.5, respectively there was a relatively wide size distribution obtained for both samples. The zeta potential data observed for the AuNPs supports the agglomeration data obtained by DLS. The low zeta potential of the Au_30nm_ sample (−4.81 mV) equates to reduced electrostatic repulsion of particles and agglomerates in the colloid, thus giving rise to the overall larger agglomerates measured in the DLS analysis. In comparison, the SiO_2_ nanoparticles displayed a hydrodynamic diameter of 68.1 nm, thus indicative of a substantially better dispersed sample as the agglomerate size is two-to-three SiO_2_ nanoparticles in diameter (based on transmission electron microscopy [TEM] primary particle size measurements). Similarly, the BaSO_4_ DLS analysis appears to be detecting single nanoparticles, whereas the CeO_2_ showed notable agglomeration in complete culture media [14].

### ENP mutagenicity evaluation

TK6, HepG2, V79, whole blood, and buffy coat cells were independently exposed to each test ENP to determine their cytotoxic and genotoxic potential over a period of one cell cycle. Cell viability and mutagenicity were then determined using RPD and CBPI, combined with an evaluation of the frequency of binucleated cells containing micronuclei.

**Figure 1.**
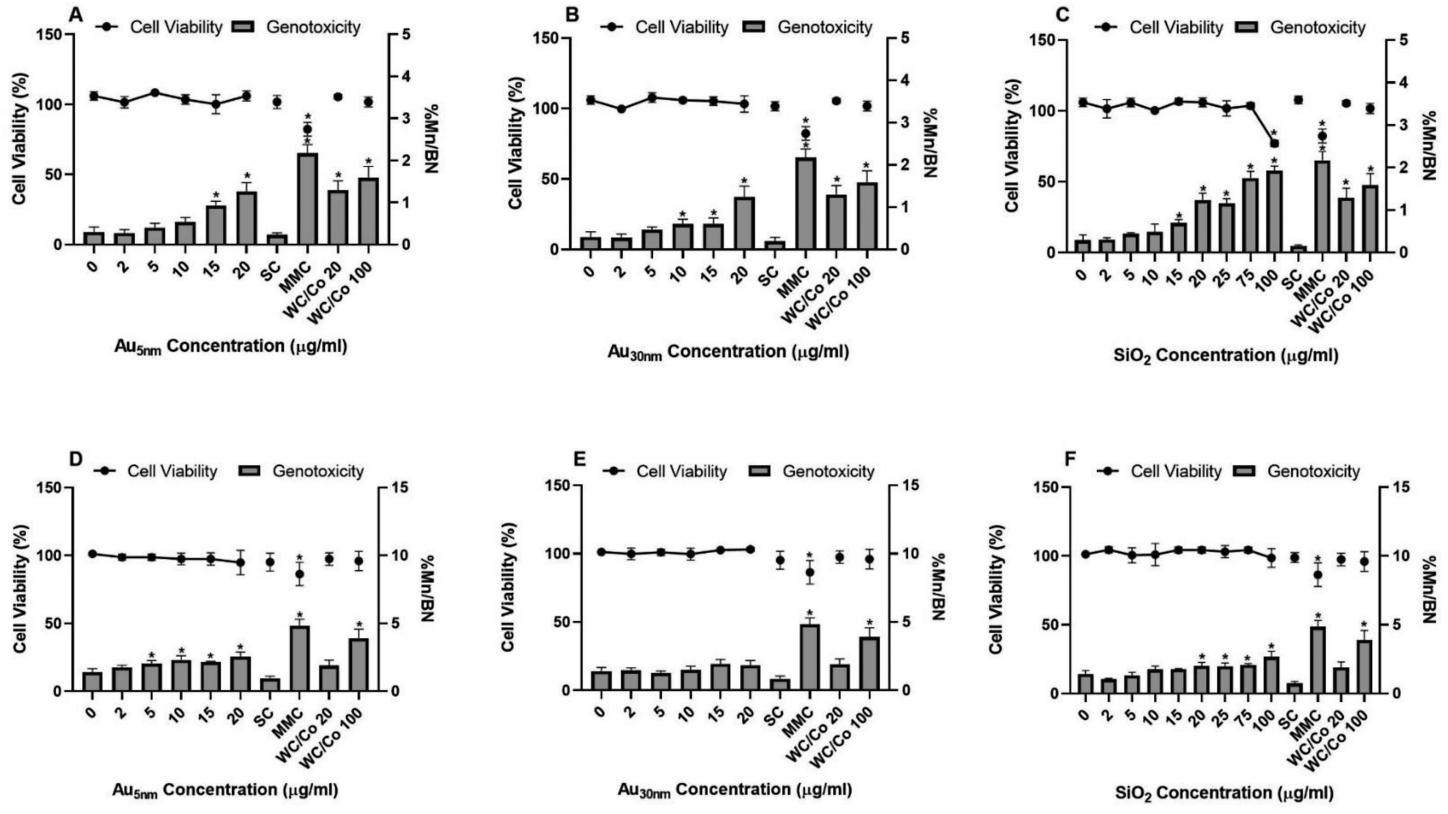
TK6 and HepG2 cell viability and mutagenicity (percentage of binucleated cells containing micronuclei (%Mn/BN)) determined by the *in vitro* CBMN assay following exposure to Au_5nm_, Au_30nm_, and SiO_2_ (TK6; A, B, C, respectively and HepG2; D, E, F). Positive controls: Mitomycin C (MMC) at 0.01 µg/ml and tungsten carbide-cobalt (WC/Co) at 20 and 100 µg/ml. Double-distilled water was used as the solvent control (100 µl). These data were generated in laboratory 1, where the results were considered statistically significant (*) when *P* ≤ .05 (*n* = 3).

The TK6 cells showed no relevant (55 ± 5% cytotoxicity) cytotoxic response to both AuNPs up to 20 µg/ml (the highest test concentration possible from the supplied stock sample). The only cytotoxic responses observed for the TK6 cells were induced by the SiO_2_ NPs at the highest test concentration of 100 µg/ml and for cells exposed to the positive chemical control MMC at 0.01 µg/ml. The Au_5nm_ ENPs significantly (*P* ≤ .05) induced micronuclei at 15 µg/ml and 20 µg/ml in a concentration-dependent manner showing 2-fold increases over background levels (0.3% (%Mn/BN)), Figure 1A. Similarly, TK6 cells when exposed to (10, 15, and 20 µg/ml) Au_30nm_ ENPs also produced significant mutagenicity (Fig. 1B), albeit a sub-two-fold increase of mutagenicity at the highest concentration of 20 µg/ml. TK6 cells exposed to the SiO_2_ ENPs showed significant increase of micronuclei at 15 µg/ml (lowest observed genotoxic effect level [LOGEL]) which then increased to 6-fold over background levels with concentrations up to 100 µg/ml (Fig. 1C). The WC/Co induced a 4- and 5-fold increase of micronuclei at concentrations of 20 µg/ml and 100 µg/ml, respectively. The positive control (MMC, a small molecule) produced significant increase of micronuclei in TK6 cells with a 6-fold increase over background levels.

The HepG2 cells showed no relevant cytotoxic response to any of the JRC reference ENPs. The only cytotoxic responses observed for the HepG2 cells was observed following MMC exposure at 0.01 µg/ml. The Au_5nm_ induced statistically significant increase in micronucleus frequency at concentrations between 5 µg/ml and 20 µg/ml with a sub-2-fold increase over background (Fig. 1D). Interestingly, the Au_30nm_ had no effect on HepG2 cells (Fig. 1E), whereas HepG2 cells exposed to SiO_2_ ENPs did show significant increases in micronuclei levels. The LOGEL was 20 µg/ml with a relatively small increase of 1.7-fold over background levels at 100 µg/ml (Fig. 1F). At the highest tested concentration of 100 µg/ml, the WC/Co induced a similar fold-change (2.7-fold) over the background as the chemical control MMC (3-fold), indicative of its potential for yielding a strong genotoxic response as a particle control.

**Figure 2.**
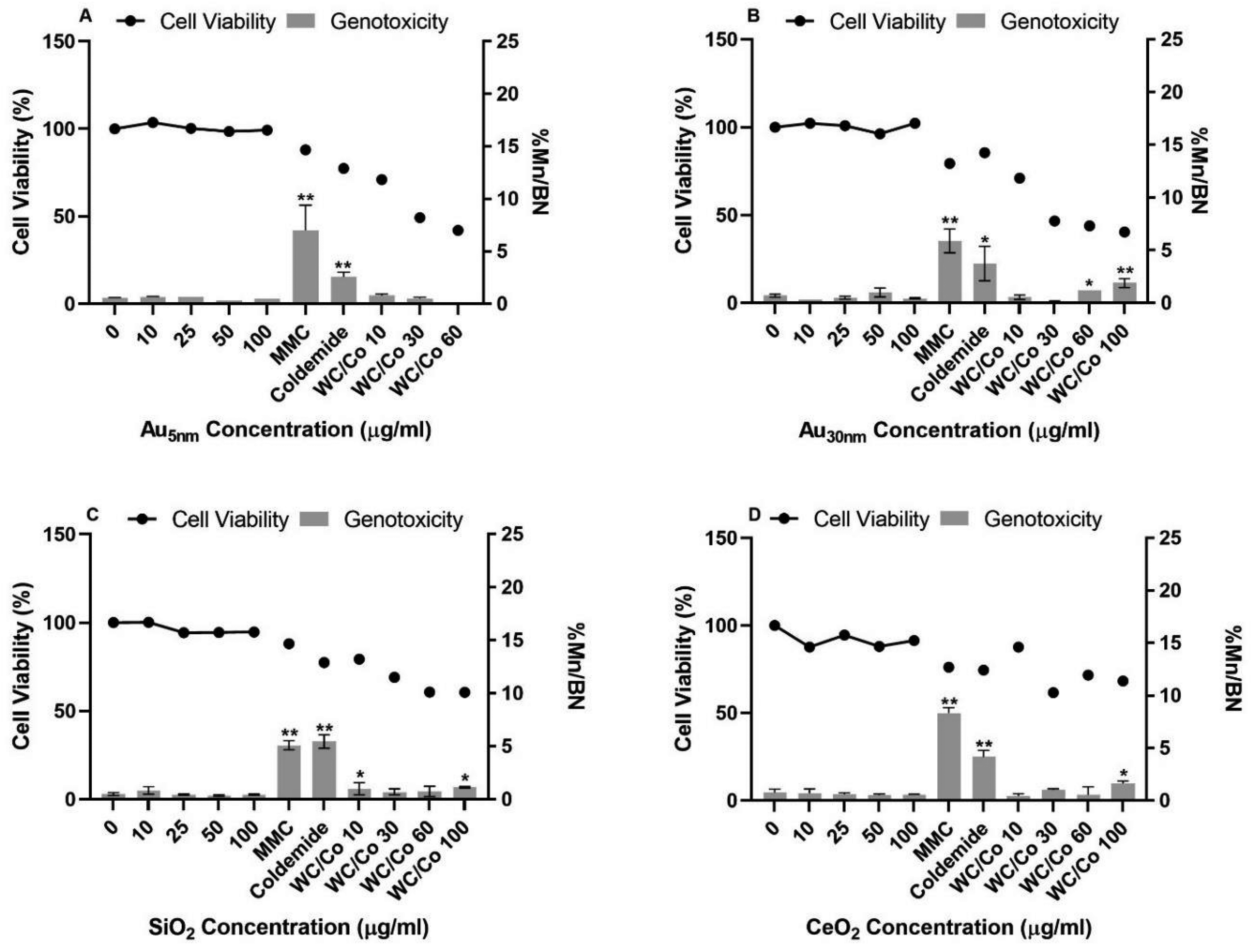
Buffy coat CBPI and frequency of micronuclei (percentage of binucleated cells containing micronuclei (%Mn/BN)) determined by the *in vitro* CBMN assay following 1 cell cycle exposure to Au_5nm_, Au_30nm_, SiO_2_, and CeO_2_ ENPs (A, B, C, and D, respectively). Positive controls used were Mitomycin C (MMC) at 0.04 µg/ml, Colcemid at 0.05 µg/ml, and tungsten carbide-cobalt (WC/Co) at 10, 30, 60, and 100 µg/ml. These data were generated in laboratory 2, where the results were considered statistically significant (*) when *P* ≤ .05 or (**) when *P* ≤ .01 (*n* = 2).

The test nanoparticles did not exert any relevant cytotoxicity in the buffy coat cell exposures. No statistically significant induction of micronuclei in binucleated cells were observed in buffy coat cells following exposures to Au_5nm_, Au_30nm_, SiO_2,_ or CeO_2_ (Fig. 2). The respective vehicle control values ranged between 0.5% and 0.8%, which was within the 95% control limit of the laboratories historical control database (micronucleus frequency: 0.2–0.9%).

The particulate control, WC/Co, tested at 10–100 µg/ml showed a concentration related increase in cytotoxicity, with a maximum of 59% at 100 µg/ml. The micronucleus counts ranged between 1.2% and 1.9%. These values are higher than the upper limit of the 95% control of the historical control data and statistically significant at least one test concentration of WC/Co as compared to the corresponding vehicle control. The two chemical positive controls MMC and colcemid, induced statistically significant increases in micronuclei ranging between 5.1% and 9.7% for MMC, and 2.3–5.5% for colcemid.

**Figure 3.**
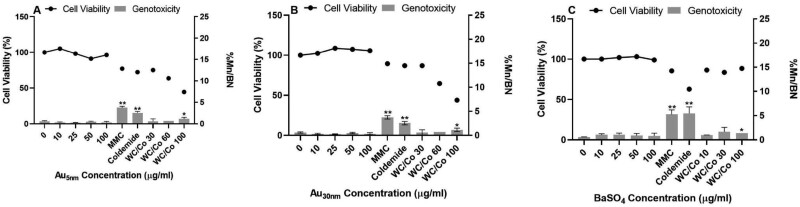
Whole blood CBPI and frequency of micronuclei (percentage of binucleated cells containing micronuclei (%Mn/BN)) determined by the *in vitro* CBMN assay following 1 cell cycle exposure to Au_5nm_, Au_30nm_, and BaSO_4_ ENPs (A, B, and C, respectively). Positive controls used were Mitomycin C (MMC) at 0.04 µg/ml, Colcemid at 0.05 µg/ml, and WC/Co at 10, 30, 60, and 100 µg/ml. These data were generated in laboratory 2, where the results were considered statistically significant (*) when *P* ≤ .05 or (**) when *P* ≤ .01 (*n* = 2).

In whole blood exposures, none of the nanoparticles significantly increased the micronuclei (statistically) as compared to the concurrent vehicle control (Fig. 3). The respective vehicle control ranged between 0.4% and 0.6%, which was within the 95% control limit of the laboratories historical control data base (micronucleus frequency: 0.2–0.9%). The positive particulate control WC/Co tested at 10–100 µg/ml showed a concentration-related increase in % cytotoxicity, with a maximum value of 56% observed. The micronucleus frequencies at the highest concentration of WC/Co (100 µg/ml) ranged between 1.2% and 1.4%. These were higher than the upper limit of the 95% control of the historical control data and statistically significant as compared to the corresponding vehicle control. The two molecular positive controls (MMC and colcemid) induced statistically significant increases in the micronuclei frequencies ranging between 3.1–5.1% for MMC and 2.2–5.5% for colcemid.

**Figure 4.**
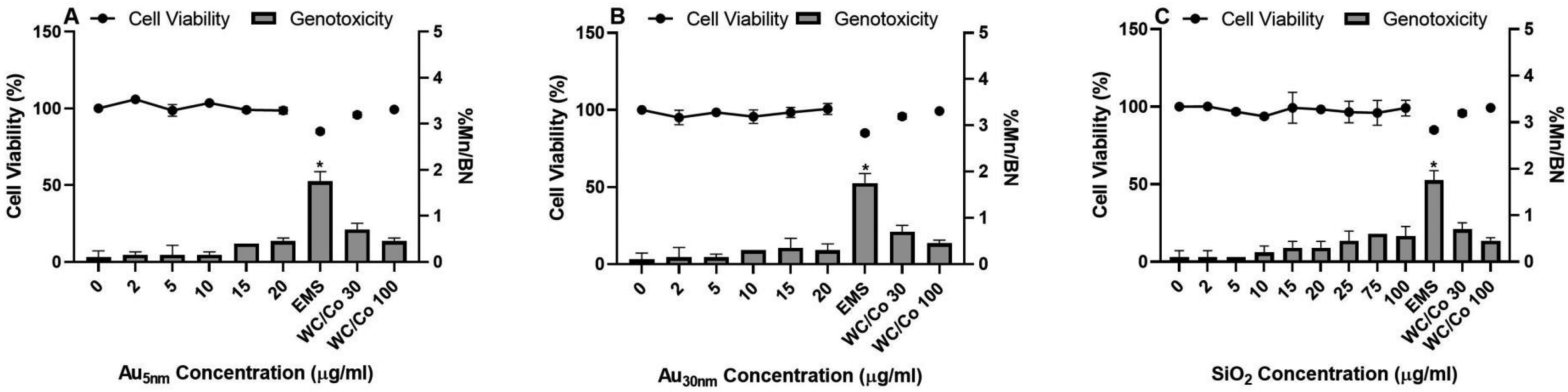
V79 CBPI and frequency of micronuclei (percentage of binucleated cells containing micronuclei (%Mn/BN)) determined by the *in vitro* CBMN assay following 1 cell cycle exposure to Au_5nm_, Au_30nm_, and SiO_2_ ENPs (A, B, and C, respectively). Positive controls used were ethyl methanesulfonate (EMS) at 600 µg/ml and tungsten carbide-cobalt (WC/Co) at 30 and 100 µg/ml. These data were generated in laboratory 1, where the results were considered statistically significant (*) when *P* ≤ .05 (*n* = 2).

**Figure 5.**
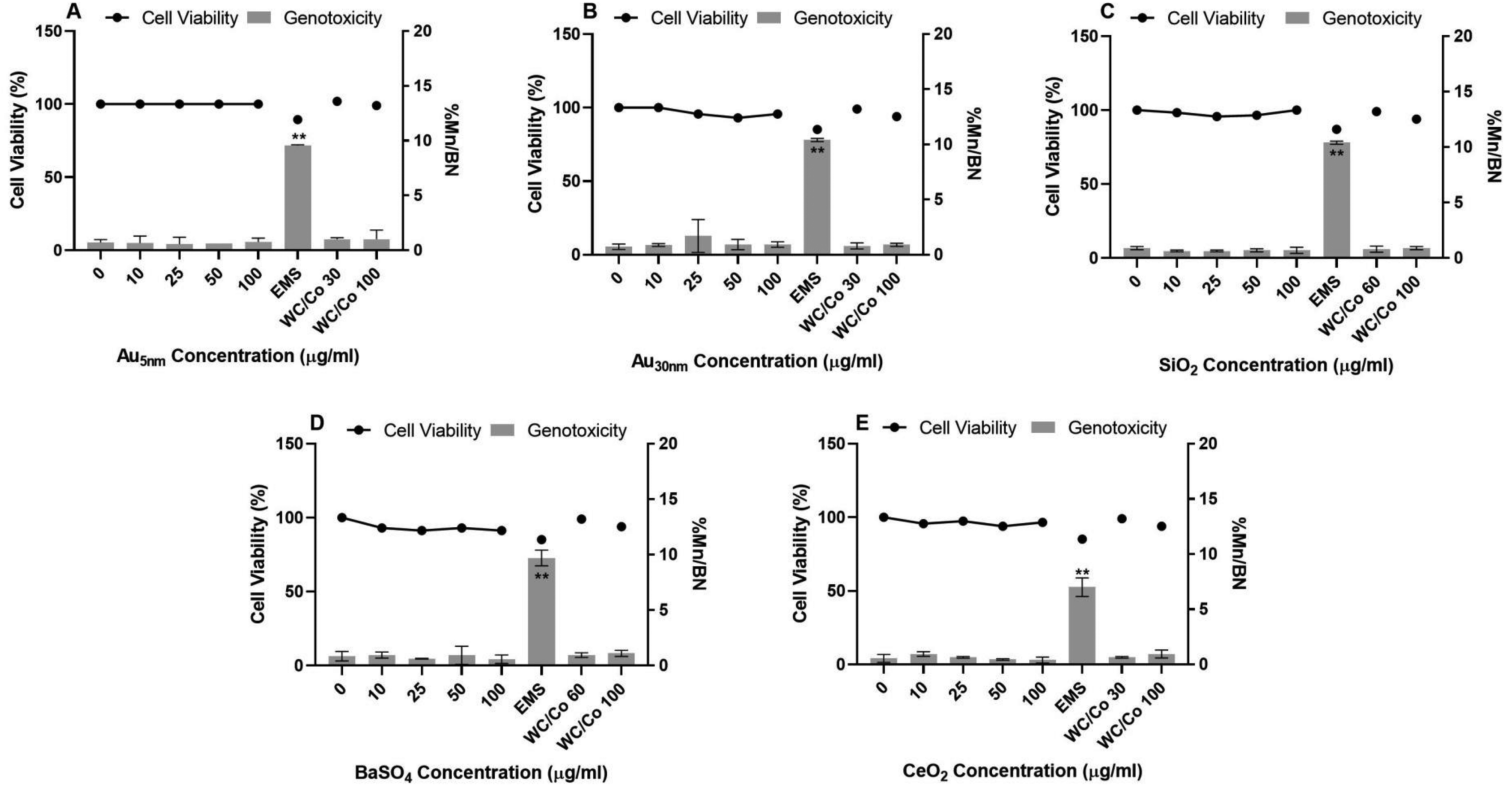
V79 CBPI and frequency of micronuclei (percentage of binucleated cells containing micronuclei (%Mn/BN)) determined by the *in vitro* CBMN assay following 1 cell cycle exposure to Au_5nm_, Au_30nm_, SiO_2_, BaSO_4_, and CeO_2_ ENPs (A, B, C, D, and E, respectively). Positive controls used were ethyl methanesulfonate (EMS) at 600 µg/ml and tungsten carbide-cobalt (WC/Co) at 30 and 100 µg/ml. These data were generated in laboratory 2, where the results were considered statistically significant (*) when *P* ≤ .05 (*n* = 2).

The V79 cells showed no relevant cytotoxic or genotoxic responses to any of the test ENPs following a one cell-cycle exposure. This cell line was evaluated by both laboratories with four test materials (detailed in Supplementary Table 1), and the results obtained were consistent across both laboratories, demonstrating inter-laboratory reproducibility (Figs 4 and 5). The positive molecular control (EMS at 600 µg/ml) induced a significant increase in micronuclei following treatment, producing a 17-fold increase over the vehicle control in binucleated cells. The genotoxic responses for each material has been summarized in Table 2.

**Table 2.** Overview of the results obtained from the genotoxicity study performed across two laboratories for each reference material.

Material	Material role in experiment	TK6	HepG2	V79	Human whole blood	Buffy coat cells
EMS MMC Colcemid	Molecular positive control	+	+	+	+	+
WC/Co	Proposed particulate positive control	+	+	−	+	+
SiO_2_	Reference nanoparticle	+	+	−	−	−
Au_5nm_	Reference nanoparticle	+	+	−	−	−
Au_30nm_	Reference nanoparticle	+	−	−	−	−
BaSO_4_	Reference nanoparticle	n.t.	n.t.	−	−	n.t.
CeO_2_	Reference nanoparticle	n.t.	n.t.	−	−	−

A positive genotoxic response is denoted as (+) and a negative genotoxic response denoted as (−), materials which were not tested with a specific cell line are denoted as (n.t.).

### Cellular concentration of ENPs

Prior to this study, the JRC published in 2020 a report on the cytotoxicity of the reference ENPs and their cell uptake capacity in several mammalian cell lines, including V79 and TK6. The JRC technical report demonstrated by TEM that both TK6 and V79 cells successfully internalized Au_5nm_ (20 µg/ml), Au_30nm_ (20 µg/ml), and SiO_2_ (100 µg/ml) [10].

In the present study, cellular ENP concentration was estimated for V79, whole blood, and buffy coat cells with LA-ICP-MS. A semi-quantitative approach in combination with single spot analysis allowing for high-throughput and providing low detection limits [17]. Phosphorus was taken as internal standard as it occurs in a variety of cellular macromolecules including membrane proteins and the DNA. The ratio of the respective elements Au, Ce, Ba, and Co to phosphorus is given as a semi-quantitative measure for the association of the tested ENPs with the cells. The data given in Table 3 show that the ratio of the individual element to the intracellular phosphorus increased concentration relatedly in each individual experiment. It must be considered that it is not possible to distinguish between particles that adhere to the cell surface and particles that have been internalized or compare the ratios between different elements due to element-specific response factors. However, the ratios between the cell type used can be compared. Thus, it can be observed that V79 cells, in all cases, have a higher ratio of the element to their intracellular phosphorus levels as compared to buffy coat or whole blood cells. This suggests a greater ENP-cellular association in V79 cells (which coincides with the findings of the JRC published report showing V79 capacity for internalizing all test particles (via TEM); however, this does not necessarily equate to a greater capacity for V79 cells to internalize ENPs [10].

**Table 3.** The ratio of the indicated elements to phosphorus in buffy coat, whole blood, and V79 cells after treatment with the indicated concentrations of the given ENPs.

µg/ml	Buffy coat cells				Whole blood cultures				V79				
	Au_5nm_	Au_30nm_	CeO_2_	WC/Co	Au_5nm_	Au_30nm_	BaSO_4_	WC/Co	Au_5nm_	Au_30nm_	CeO_2_	BaSO_4_	WC/Co
	Au/P	Au/P	Ce/P	Co/P	Au/P	Au/P	Ba/P	Co/P	Au/P	Au/P	Ce/P	Ba/P	Co/P
0	<LOD	<LOD	<LOD	<LOD	<LOD	<LOD	0.02	<LOD	<LOD	<LOD	<LOD	0.03	<LOD
10	0.01	0.003	0.04	--	0.008	0.01	--	--	0.23	0.46	57.53	--	--
25	--	--	--	--	--	--	--	--	--	--	--	--	--
30	--	--	--	0.08	--	--	1.24	0.019	--	--	--	27.32	0.45
50	0.02	0.010	0.36	--	0.035	0.03	--	--	0.48	2.01	219.86	--	--
60	--	--	--	0.1	--	--	1.61	0.045	--	--	--	32.53	0.95
100	0.04	0.058	3.81	0.16	0.089	0.16	1.97	0.072	0.76	4.56	334.19	35.71	1.26

## Discussion

This study evaluated the cytotoxic and genotoxic potential of Au_5nm_-, Au_30nm_-, SiO_2_-, CeO_2_-, BaSO_4_ -NPs, and WC/Co ENMs in TK6, HepG2, V79, whole blood, and buffy coat cells using a harmonized nano-specific protocol for the *in vitro* CBMN assay. The experiments were conducted in two independent laboratories to provide evidence to support the development of an OECD guidance document, which is necessary for adapting the experimental approach outlined in the OECD TG No. 487 for the evaluation ENPs.

Beside the influence of size, surface charge, and functionalization on the cellular uptake of ENPs, the experimental design is also assumed to have a strong impact on the ENP-cell interaction [18–20]. To explain potential differences in the cytotoxicity and mutagenicity potential of metallic ENPs, their uptake into the cells was monitored. Due to its excellent sensitivity for nearly all elements of the periodic system, ICP-MS is a key technique for the determination of the cell exposure of metal-based NPs [21]. The combination of ICP-MS with laser ablation (LA) enables a direct analysis of cells and tissues on a microscopic slide minimizing the time for sample preparation [22]. For a (semi-) quantitative analysis as applied in this study, different calibration, and normalization strategies, including external calibration with dried pL-droplets and internal standardization approaches are applied each proving certain advantages and limitations [23,24]. Here, we used a semi-quantitative approach in combination with single-spot analysis. The latter is suitable for a high throughput and provides low detection limits [17]. Although the method provides dose information on the ENP association to the cell, there is no direct comparability between different materials (this issue can be solved by the application of a calibration strategy). Even though the species information and potential particle surface transformation cannot be determined using an ICP-MS, LA-ICP-MS in single-particle mode enables the determination of the particle size and provides insight into the dissolution or aggregation of nanoparticles [25].

### Recommendations for the dispersion of engineered nanomaterials

The dispersion protocol used prior to administering ENPs under *in vitro* conditions, particularly submerged conditions may affect their toxicity [26]. A multitude of sonication and dispersion approaches are used in laboratories. ENPs are supplied by a manufacturer as either a dry powder or in a stable suspension. The latter can be directly used or only requires vortexing or sonication following the manufacturer’s guidance. However, it is common to receive ENPs as dry powders. These materials must first be suspended in a suitable buffer prior to sonication utilizing a suitable protocol.

Through several European Horizon 2020 projects, efforts have been undertaken to harmonize suspension protocols, including the NANoREG protocol [12]. Ultimately, this allows *in vitro* data, in particular dose–response relationships, to be compared across different laboratories. What is essential prior to toxicological testing is that the ENP suspension is thoroughly dispersed and stable. Other dispersion techniques are available, for example, the DeLoid protocol, which offer detailed information on dispersing ENPs and incorporating agglomerate analysis and dosimetry calculations [27]. The impact of sonication upon the modulation of ENP toxicity has been explored in detail by numerous studies, which have highlighted the effects of sonication time, acoustic energy delivered, and suspension buffers [28–33]. In the present study, the reference ENPs provided by the JRC were already in a stable suspension and thus only a brief sonication in a water bath was required to ensure the samples were thoroughly mixed prior to exposures. The WC/Co, CeO_2_, and BaSO_4_ were provided as powders and were suspended using the NANoREG protocol. As detailed in the report published by the JRC the polydispersity index recorded for Au_5nm_, Au_30nm_, and the SiO_2_ ENPs was 0.21, 0.25, and 0.06, respectively which can be considered monomodal (PDI of 0-0.1) to moderately polydisperse (PDI > 0.1) [9]. As seen in Table 1, PDI values for WC/Co being 0.76 and BaSO_4_ PDI of 1 typically represents a truly polydisperse sample.

### Recommendations for exposure conditions

The present study was built upon previous recommendations for how best to adapt the *in vitro* micronucleus assay for evaluating ENPs [1,5]; these recommendations were implemented by establishing a harmonized operating protocol that was subsequently evaluated across two laboratories. Diffusion and uptake by cells are usually slower for ENPs than for dissolved molecules. Hence, ENP *in vitro* testing must include adequate exposure time. The current OECD TG No. 487 specifies that cells should be treated for 3–6 hours (pulse) as well as a period corresponding to 1.5–2.0 cell cycles and sampled at a time equivalent to 1.5–2.0 cell cycles after exposure/treatment [13]. Cellular division and progression through mitosis is a crucial point during the exposure period, as the nuclei division process provides a unique opportunity for ENP to potentially come into direct contact with the DNA molecule, allowing evaluation of primary-direct DNA damage where relevant [15,34]. The nuclear uptake of ENPs is hampered by the nuclear envelope. Generally, only very small ENPs with primary diameters of ~2 nm have the potential for nuclear uptake, and even then, this process is material-specific [35,36]. Differences in the literature concerning the exposure time of cell lines to various ENPs can make it difficult to perform read-across and grouping meta-analysis. Therefore, it would be highly beneficial to mitigate these differences by unifying exposure time to 1–1.5 cell cycle(s) to, in essence, normalize ENP exposure across the field.

The capacity of ENPs to be internalized by cells *in vitro* is correlated to their physico-chemical features such as size and shape. Generally, smaller materials with spherical geometries could facilitate cellular uptake with greater efficiency when compared with cuboidal structures and fibrous materials [37,38]. It has been demonstrated that ENP uptake is dependent on several factors: cell type, composition, shape, dose, time, state of agglomeration, etc. [39]. As suggested before, a 1–1.5 cell cycle, provides sufficient time for cellular uptake of the material [1,5,40,41]. Along with the impact of ENP exposure time, the period of incubation with cyto B is crucial. The recommendation is a 1.5 cell cycle time with cyto B, which is applied post-exposure, i.e. following completion of the exposure period with the test agent and after washing of the cells. At present, the OECD TG No. 487 recommends the co-incubation of the test substance with cyto B. However, the guideline does state that in cases where the presence of cyto B may interfere with the exposure to the test substance, the exposure period maybe prolonged by a further 1.5–2 cell cycles. In case of ENP, it is crucial that the cyto B be added following the exposure period; thus, the cells have had sufficient time to internalize the test material. Cytochalasin B has been shown to interfere with actin filaments required for endocytosis and thus co-exposures should be avoided; this recommendation have been extensively outlined in previous publications [1,5,42]. Recently, Fernandez-Bertolez (2022) have reported that cellular uptake of TiO_2_ ENPs and micronucleus induction is not affected by the presence or absence of cyto B during the exposure period in SH-SY5Y cells [43]. This could indicate that in some cells other modes of nanomaterial uptake is present. However, since the separation of the exposure period to an ENP and the cyto B treatment phase does not have a detrimental effect on the outcome of the results, it still is recommended not to treat the cells with the test substance and cyto B in parallel.

The OECD TG No. 487 specifies that ENPs should not induce more than 55% ± 5% cytotoxicity. However, in situations—as within the present study—where no cytotoxicity is observed, it is recommended that the highest concentration of ENPs does not exceed 100 µg/ml or 100 µg/cm^2^. This avoids excessive variability in agglomeration dynamics and minimizes the use of doses that are not physiologically relevant; moreover, the *in vitro* dose can be correlated to *in vivo* doses by established extrapolation methods [44].

Dose-dependent changes in the agglomeration dynamics of ENPs have been investigated in the work by Wills *et al*. in 2017 [45]. The authors of the paper refer to ‘tipping points’, which are introduced when high concentrations of ENPs are delivered to cells *in vitro* under physiological conditions, ultimately effecting the biological responses. Typically, at high doses, agglomeration is more predominant with reduced dispersion stability than at lower doses when a stable dispersion of the particles can be more readily achieved. This results in cellular uptake variation across a dose range, and thus, a dose-dependent genotoxicity response will not be likely for all ENMs. This has previously been highlighted by Murugadoss *et al*. (2020), whereby the agglomeration of TiO_2_ ENPs can induce significantly different responses to a well-dispersed suspension, ultimately influencing the observed toxicological responses both *in vitro* and *in vivo* [46]. Thus, excessive agglomeration of ENPs (a consequence of inadequate dispersion) can result in decreased cellular uptake, which may correlate with less DNA damage being observed.

### The use of positive controls

Currently, there are no agreed particulate positive controls for mutagenicity assays. Therefore, molecular positive controls are used. Positive chemical controls should induce a statistically and biologically significant increase over untreated and/or solvent control background levels when conducting the *in vitro* CBMN assay [15,16,47–51]. The present OECD TG No. 487 stipulates that the positive control produce reproducible, and detectable increases over background levels to demonstrate the sensitivity of the test system, however the effects must not be compromised by the concentration of the positive control exceeding the cytotoxicity limits specified by this guidance [13].

While there are no established particulate control materials, WC/Co ENPs may prove valuable, which has been alluded to in the prior studies by Moche *et al.* in 2014. They evaluated the mouse lymphoma, alkaline comet, and micronucleus assays with L5178Y and primary human lymphocytes, which were exposed to WC/Co ENPs up to 120 µg/ml. Moche *et al*. reported statistically significant mutation frequency after 4-hour (80 µg/ml) and 24-hour (35 and 90 µg/ml) exposures in the mouse lymphoma assay. Using the mononucleate micronucleus assay in L5178Y cells, the authors similarly reported statistically significant levels of micronuclei at 4 hours (80 and 100 µg/ml) and 24 hours (80 µg/ml). Moche and colleagues also demonstrated that the WC/Co induced highly significant micronuclei frequency in primary human lymphocytes at 4 hours (80 µg/ml) and 24 hours (20, 40, 60, and 80 µg/ml). These findings were supported by significant DNA damage detected using the alkaline comet assay which showed a greater elevation of DNA damage after 4 hours of exposure [52]. TK6 cells in the present study showed the greatest sensitivity to WC/Co with > 2-fold increases over background levels of micronuclei frequencies at concentrations of 20 and 100 µg/ml. The buffy coat cells, nevertheless, showed statistically significant, reproducible increases in the micronucleus numbers above the historical control data, whereby the increases observed in buffy coat cells were more prominent than those from whole blood cultures. This could most probably be due to a higher exposure of the target lymphocytes to the ENMs in the absence of the interfering erythrocytes. Cell-specific mutagenicity was observed in both laboratories and thus agrees with the conclusions drawn by Moche *et al*. in 2014 and 2015, which stated the use of WC/Co as a particulate control may be cell line specific [52,53]. In the 2015 paper published by Moche *et al*., the authors undertook a mechanistic approach to elucidating the DNA damage conducting chromosome aberration assays, kinetochore staining of micronuclei and oxidative stress assessments. The number of micronuclei both, containing and lacking centromeres was approximately the same in both cell types tested, the L5178Y and human lymphocytes. In the present study, there was a significant trend towards centromere negative micronuclei at the highest concentration of WC/Co, 100 µg/ml in TK6 cells (Supplementary Data). Nevertheless, the combined research of the 2014 and 2015 papers by Moche *et al*., and the present study has highlighted WC/Co as a genotoxic material and particle control only in specific cell types; the evidence is currently lacking to recommend it as generally applicable to all test cells. The suspension cells appeared to be more sensitive to induced ENP damage, with TK6 and buffy coat cells demonstrating significant DNA damage following exposure to the WC/Co, whereas the adherent cells did not.

### The test system

The optimal test system to be used for ENP testing should provide a high degree of sensitivity towards the detection of genotoxic effects [5]. Nevertheless, it should also reflect the physiologically relevant situation, thus minimizing false positive results which trigger follow up studies *in vivo* [5]. This is a special challenge for ENP testing due to the scarcity of reliable *in vivo* data for use as a benchmark. In this study, two ENPs (BaSO_4_ and CeO_2_) were tested negative using buffy coat cells. Both ENPs have been shown to be non-carcinogenic via the inhalation route of exposure [44]. The most appropriate test system could not be identified in this study. In general, suspension cells provided more reproducible results compared to the adherent cells. Adherent and suspended cells responded similarly to the soluble positive control but had contradictory responses to the particulate positive controls. Exposure was demonstrated in both TK6 cells and buffy coat cells. However, at present, no certain explanation can be given for the discrepancy between the results obtained for TK6 cells and buffy coat cells. Considering that oxidative processes might be a mechanism for the ENP induced mutagenicity, an explanation for the different results might be the inherent capacity of the individual cell types to cope with oxidative stress. However, this needs to be addressed in follow up studies.

Another aspect, which has not been dealt with in this study is the use of metabolic activation such as S9 mix. It is generally assumed that the use of metabolic activation on inorganic ENPs is redundant. However, the question remains open on the application of metabolic activation for organic ENPs. The use of S9 mix introduces a new challenge to the protocol introduced in this study, namely the recommendation of an exposure period of 1 cell cycle is not compatible in the presence of S9 mix, due to its cytotoxicity. This can be circumvented by a washing step after 4 hours. Since the ENPs remain in the culture of suspension cells, the treatment phase can be continued for a further 20 hours prior to the addition of cyto B.

## Recommendations and Conclusions

A harmonized nano-specific protocol has been developed for the *in vitro* micronucleus assay, which was evaluated by testing a total of six ENPs on five different cell lines. Additionally, one cell line was evaluated with four ENPs by two laboratories, and the resultant datasets were concordant, demonstrating inter-laboratory reproducibility. This study has highlighted several crucial factors that must be implemented to facilitate future nano-safety testing using this test, including:

- It is important that the most sensitive cell system is used; this includes a cell that is preferably of human origin and p53 competent, with a stable and suitably low background micronucleus frequency.
- The dispersion technique must be appropriately selected to ensure the test material is stable, and thoroughly suspended with supporting characterization data of at least one of the test concentrations (but ideally multiple concentrations across the test range). Dispersion characterization at the time of exposure and post-exposure is recommended.
- The maximum concentration for ENP testing should be 100 µg/ml to maintain physiological relevance and minimize agglomeration.
- The minimum exposure time should be one cell cycle (which will vary between cell lines), followed by a 1–1.5 cell cycle incubation with cyto B, having first removed the exposure media and washed the cells thoroughly.
- Cellular uptake/internalization of the ENP should be evaluated and is of particular importance when negative results for the mutagenicity assays have been observed, as this information is required to determine if a test material is truly non-genotoxic or if the material did not reach the target cell. It is important to note that some materials may induce genotoxicity in the absence of uptake due to dissolution; this is particularly the case for those materials that consist of metal components or contaminants. In such cases, dissolution studies may also add significant value to mutagenicity studies when interpreting data, but technical challenges remain when evaluating this parameter.
- Cells should be exposed to a suitable chemical and particulate positive control which produces a consistent and reproducible increase in micronuclei when compared with the vehicle control (under blinded conditions).

The data generated in this study confirm that ENP cytotoxicity and mutagenicity can be tested with the adapted protocol of OECD TG No. 487. The suspension cells (TK6, whole blood, and buffy coat cells) proved to be most sensitive to mutagenicity induction with a range of ENPs. Furthermore, WC/Co induced a reproducible, statistically significant increase in genotoxicity following exposure to TK6 cells, whole blood, and buffy coat cells. However, there remains insufficient evidence of this being a generally applicable positive particulate control as no substantial induction of micronuclei was observed in V79 cells exposed to WC/Co.

Implementing the recommendations above provides an adapted protocol for the evaluation of ENP genotoxicity using the *in vitro* micronucleus assay, addressing several key issues in the required experimental approach. This harmonized protocol has been assimilated into an OECD Guidance Document: ‘Study Report and Preliminary Guidance on the Adaptation of the *In Vitro* micronucleus assay (OECD TG 487) for Testing of Manufactured Nanomaterials’, published by the OECD in Sept 2022 (Series on Testing and Assessment No. 359; ENV/CBC/MONO(2022)15).

## Supplementary data

Supplementary data is available at *Mutagenesis* Online.

geae010_suppl_Supplementary_Materials

## Data Availability

The data generated in this manuscript are available from the corresponding author upon reasonable request.
