# Supplementary material for: Adapting the in vitro micronucleus assay (OECD Test Guideline No. 487) for testing of manufactured nanomaterials: recommendations for best practices
Source: Mutagenesis. 2024 Mar 19;39(3):205–17. doi: 10.1093/mutage/geae010 (PMC11040148; doi:10.1093/mutage/geae010)
Supplement: geae010_suppl_Supplementary_Materials [file geae010_suppl_supplementary_materials.docx]

**Supplementary Information for: Adapting the *in vitro* micronucleus assay (OECD Test Guideline No. 487) for testing of manufactured nanomaterials: recommendations for best practices**

Michael J Burgum^#1^, Clarissa Ulrich^#2^, Natascha Partosa^#2^, Stephen J Evans^1^, Caroline Gomes^2^, Svenja Berit Seiffert^3^, Robert Landsiedel^2,4^, Naveed Honarvar*^2^, Shareen H Doak*^1^

1. In Vitro Toxicology Group, Faculty of Medicine, Health and Life Sciences, Institute of Life Sciences, Swansea University Medical School, Singleton Park, Swansea, SA2 8PP, Wales, UK .
2. BASF SE, Experimental Toxicology and Ecology, 67056, Ludwigshafen Germany
3. BASF SE, Analytical and Material Science, 67056, Ludwigshafen Germany
4. Free University of Berlin, Pharmacy – Pharmacology and Toxicology, 14195 Berlin, Germany

#Joint first authors contributed equally to this manuscript

*Corresponding Author 1:

# Tel: +44 1792 295388

E-mail address: s.h.doak@swansea.ac.uk

*Corresponding Author 2:

# Tel: +49 621 60 58057

Fax: +49 621 60 51734

E-mail address: [Naveed.honarvar@basf.com](mailto:Naveed.honarvar@basf.com)

**Methods**

***Kinetochore staining of micronuclei***

To determine if the DNA damage was a consequence of an aneugenic or clastogenic response,

kinetochore staining of micronuclei in BN cells was performed. Following the exposure period, the cells were washed three times with PBS before being cytocentrifuged (500 g, 5 minutes) onto microscope slides and fixed in ice-cold 90% methanol at −20 °C. Immunofluorescent staining of kinetochore proteins was performed as previously described (15, 16). Kinetochore scoring was performed on a Zeiss AxioCam HRc (Carl Zeiss Microscopy and Imaging, UK). Only the lowest and highest significant ENM concentrations were used for each particle type, with WC/Co included at 100 µg/mL, of which 50 micronuclei were scored for the presence or absence of FITC fluorescence in the micronucleus indicating the presence of a whole chromosome (K+) or chromosome fragment (K-) respectively.

**Supplementary Table 1. Breakdown of the ENPs tested by which laboratory and in which cell lines.**

|  | **ENP** | | | | | |
| --- | --- | --- | --- | --- | --- | --- |
|  | **Au_5nm_** | **Au_30nm_** | **SiO_2_** | **WC/Co** | **CeO_2_** | **BaSO_4_** |
| **TK6** | Laboratory 1 | Laboratory 1 | Laboratory 1 | Laboratory 1 |  | |
| **HepG2** | Laboratory 1 | Laboratory 1 | Laboratory 1 | Laboratory 1 |  |  |
| **V79** | Laboratory 1 & 2 | Laboratory 1 & 2 | Laboratory 1 & 2 | Laboratory 1 & 2 | Laboratory 2 | Laboratory 2 |
| **Whole Blood** | Laboratory 2 | Laboratory 2 |  | Laboratory 2 |  | Laboratory 2 |
| **Buffy Coat Lymphocytes** | Laboratory 2 | Laboratory 2 | Laboratory 2 | Laboratory 2 | Laboratory 2 |  |

**Supplementary Table 2. Metafer microscope (Metafer 4, version 3.5) nuclei classifier settings.**

| Nuclei Parameter | Nuclei Classifier | Micronuclei Classifier |
| --- | --- | --- |
| Minimum Area | 20µm^2^ | 1.5µm^2^ |
| Maximum Area | 400µm^2^ | 55µm^2^ |
| Maximum Relative Concave Depth | 0.9 | 0.9 |
| Maximum Aspect Ratio | 1.5 | 4 |
| Maximum Distance | 30µm^2^ | 25µm^2^ |

**Results**

Supplementary Figure 1. Kinetochore staining of TK6 micronuclei in binucleated cells. The data revealed a trend towards a clastogenic response denoted by the dark grey bars of the graph as opposed to the loss of entire chromosome (light grey), biological *n*=1.
